# Influence of providing information to participants about development of trial outcomes on response rates and attitudes to questionnaire completion: Protocol for a study within a trial

**DOI:** 10.12688/hrbopenres.12895.2

**Published:** 2019-05-20

**Authors:** Charlotte Griffin, Elaine Toomey, Michelle Queally, Catherine Hayes, Patricia M. Kearney, Karen Matvienko-Sikar

**Affiliations:** 1 School of Public Health, University College Cork, Cork, T12 XF62, Ireland; 2 School of Psychology, National University of Ireland, Galway, Galway City, H91 EV56, Ireland; 3 Discipline of Economics, National University of Ireland, Galway, Galway, H91 EV56, Ireland; 4 School of Medicine, Trinity College Dublin, Dublin, D06 W226, Ireland

**Keywords:** Core outcome set, COS, study within a trial, SWAT, infant feeding, childhood obesity, response rates, questionnaire completion, outcome measurements.

## Abstract

**Background** **:** Issues with questionnaire completion introduce bias and limit examinations in trials. Improving communication with participants about trial processes, such as outcome and questionnaire development, may improve questionnaire completion and response rates. Providing information about the involvement of stakeholders in the development of core outcome sets (COS) measured in trials may improve responding by tapping into subjective norms and behaviour change mechanisms. The aim of this Study Within a Trial (SWAT) is to examine if questionnaire response rates and participants’ attitudes towards questionnaire completion are impacted by providing information about COS use in a trial of a complex intervention.

**Methods:** This is a randomised, single-blinded, parallel group intervention SWAT, embedded within a feasibility trial of an infant feeding intervention to prevent childhood obesity. The SWAT intervention consists of a brief written description and explanation about the development and use of a COS of infant feeding outcomes to prevent childhood obesity, used in the trial. Participants are parents or caregivers of infants aged two months at questionnaire completion. Participants will be randomly assigned to receive the SWAT intervention prior to questionnaire completion (SWAT Intervention), or not (SWAT Comparator). The primary outcome of interest is response rates, which will be measured as proportion of questionnaire completion and individual item response rates. Participants’ attitudes will also be assessed using closed-ended and an open-ended question to evaluate participants’ attitudes about questionnaire completion.

**Discussion:** We hypothesise that providing information about development and use of a COS will increase questionnaire response rates and attitudes toward questionnaire completion relative to the control condition. Findings will indicate the potential usefulness of this strategy for improving participant attitudes and response rates in trials.

**Trial Registration:** This SWAT is registered on the Northern Ireland Hub for Trials Methodology: Research SWAT Repository ( SWAT57).

## Introduction

Evaluation of questionnaire responses is an important dimension of the critical appraisal of health research ( Edwards *et al*., 2009). Incomplete questionnaire responses and participant attrition increase the likelihood of bias and reduce statistical power in trials through reduction of the effective sample size ( Edwards *et al*., 2009; Fewtrell *et al*., 2008; Schulz & Grimes, 2002). Further, even in well-designed studies, factors related to research management can influence participant retention and impact questionnaire response rates, leading to research waste ( Al-Shahi Salman *et al*., 2014).

A number of potential methods to effectively increase response rates have been identified, including: the use of monetary incentives, ( Brueton *et al*., 2014); telephone or postal contact with participants prior to questionnaire distribution ( Edwards *et al*., 2009); and personalising questionnaires or survey packs with participants name and/or including a hand-written signature from the principal investigator ( Sahlqvist *et al*., 2011; Scott & Edwards, 2006). However, reviews have highlighted heterogeneity among strategies used across trials (e.g. differences in the types of incentives used between studies) thus limiting synthesis and conclusions that can be drawn about effectiveness ( Edwards *et al*., 2009). As such, there remains a need to further examine strategies to improve response rates.

Improving communication with participants about aspects related to their trial participation may be one useful strategy. Such communication is posited to enhance participant engagement with research processes in ways that are meaningful to the participant ( Gillies & Entwistle, 2012). For instance, there is some evidence to suggest that participants who feel they are better informed about trial processes tend to have more favourable attitudes toward the trial and are therefore more willing to participate in the trial ( Ellis *et al*., 2001). However, participants and the general public are suggested to have a poor understanding of different aspects of health research ( Ellis *et al*., 1999). This is problematic if it influences participant attitudes and limits engagement with trial processes; there is therefore scope to improve information provision to trial participants.

In terms of enhancing communication to improve questionnaire response rates specifically, one approach may be via providing participants with information about how outcomes are chosen and/or how questionnaires are developed for use in trials. This would be particularly useful where outcomes and questionnaires are developed via engagement with expert stakeholders as is typically done in the development of Core Outcome Sets (COS)( Williamson *et al*., 2017). COSs are standardised sets of outcomes that represent the minimum outcomes that should be measured and reported in trials for a specific health area or population ( Williamson *et al*., 2012; Williamson *et al*., 2017). COSs improve evidence synthesis by reducing outcome heterogeneity and reporting risk of bias ( Williamson *et al*., 2012), which have been noted in a range of health areas, including paediatrics ( Gardner & Kelleher, 2017; Webbe *et al*., 2018), infant feeding ( Whitford *et al*., 2018), and childhood obesity ( Matvienko-Sikar *et al*., 2018; Redsell *et al*., 2016). Expert stakeholders in COS development can include patients, clinicians, trialists, researchers and the public ( Williamson *et al*., 2017). It is suggested that engagement with such stakeholders increases the likelihood that a COS will be relevant and used by these stakeholders in research and practice ( Williamson *et al*., 2017); how engagement of participant stakeholders influences subsequent participant endorsement and use of the COS has not however been fully examined. Knowledge about stakeholder involvement in COS development may influence participant attitudes and response rates via perceptions of subjective norms around the importance of trial outcomes, where stakeholders included are representatives of the participant group. Where COS development involved clinicians and/or practitioners, such stakeholders may be perceived by patient and/or public participants as representing credible sources. This may enhance participant response rates as credible sources have been identified as a useful behaviour change technique (BCT) ( Michie *et al*., 2013), for increasing trial engagement in other trials ( Nyman *et al*., 2018; Parveen *et al*., 2016; Redfern *et al*., 2016).

Providing participants with information about COS in trials serves a dual purpose by informing participants about the outcomes of importance being measured in the trial, and highlighting the role of relevant stakeholders in developing the COS being measured in the trial. The influence of informing participants about the development and use of COS in trials on their attitudes towards and completion of trial questionnaires has not yet been examined. This research posits that including information related to COS development and measurement may serve to increase participant knowledge of these processes and/or lead to more favourable attitudes toward questionnaire completion, which would subsequently increase response rates. The aim of this study is therefore to conduct a study within a trial (SWAT) ( Treweek *et al*., 2018a) to examine if provision of information regarding development of a COS influences participants’ questionnaire response rates and attitudes towards questionnaire completion.

## Methods

This SWAT is registered on the Northern Ireland Hub for Trials Methodology Research SWAT Repository ( SWAT57).

### Design

This is a randomised, single-blinded, parallel group intervention SWAT embedded within the Choosing Healthy Eating for Infant Health (CHErIsH) feasibility trial (protocol currently in preparation for submission). The CHErIsH trial involves a brief clinical intervention targeting parents and caregivers to improve infant feeding behaviours between the ages of 0–13 months, delivered during routine primary care-based vaccination visits, alongside an implementation strategy targeted at the healthcare professional (HCP) level to support the delivery of this clinical intervention.

### Study participants

Participants for the SWAT will be the parents or primary caregivers participating in the CHErIsH feasibility trial. The CHErIsH trial participants are recruited from all parents or primary caregivers of infants under 6 weeks of age attending vaccination visits with a participating GP and or practice nurse in the trial site, a primary care centre in the south of Ireland. On average, 450 infants per annum are born to parents attending the primary care centre and during the 3 month recruitment period, it is anticipated that approximately 112 of these will be eligible for recruitment. 

### The Study Within A Trial (SWAT)

The SWAT intervention is a written informational intervention, consisting of a brief written explanation about the COS used in the development of the CHErIsH feasibility trial questionnaires, including the involvement of relevant stakeholders in developing this COS. The COS used is a COS of infant feeding outcomes for inclusion in trials of infant feeding interventions to prevent childhood obesity ( Matvienko-Sikar *et al*., 2018a; Matvienko-Sikar *et al*., 2018b). The COS was developed in a four-stage process, involving expert stakeholders in the final three stages ( Matvienko-Sikar *et al*., 2017a). Expert stakeholders were parents, HCPs, researchers and childcare professionals.

**Figure 1.**
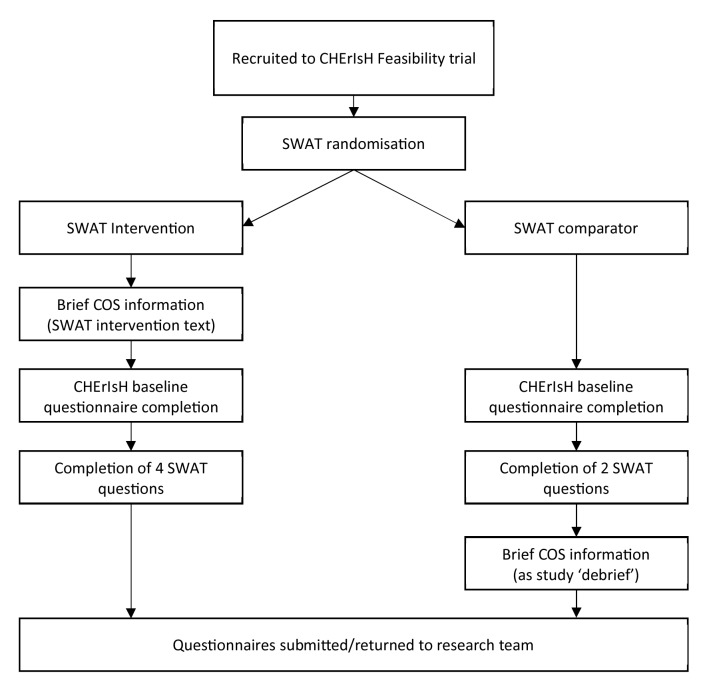
SWAT intervention flowchart.

The SWAT informational intervention will be provided to those randomised, using a random number generator, to the SWAT Intervention group; participants and will be blinded (single blind) to the group assigned ( Figure 1). In the SWAT Intervention, participants will receive the SWAT intervention, in the form of the brief COS information presented at the beginning of the CHErIsH questionnaire at trial baseline (when the infant is less than 2 months old) in the SWAT Intervention. Participants randomised to the SWAT Comparator will receive the information on the COS following completion of both the CHErIsH and SWAT questionnaires; this is to ensure all participants are provided with equal information following questionnaire completion. ( Figure 1).

Information about the COS will be provided to a random sample of half of all participants at the beginning of the CHErIsH Questionnaire in a brief paragraph including the following:

- A statement that the questionnaires include measurement of outcomes from an infant feeding COS.
- A lay-summary of what a COS is and how COSs can improve examination of trial outcomes informed by the Core Outcome Measures in Effectiveness Trials (COMET) Initiative COS lay summary ( COMET, 2018).
- A brief description of how the infant feeding COS was developed with experts, including parents of infants and HCPs.

The included SWAT Intervention text is as follows:

This questionnaire includes questions about infant feeding that were put together as part of a core outcome set. Core outcomes sets are a group of outcomes (related to questions in a questionnaire) that should be measured in all studies in a health area. They are important because they allow researchers to bring together findings from many different studies to give us a better understanding about what works and what doesn’t. This improves the quality of information and helps us develop and examine better healthcare programmes and strategies.

Parents of infants, healthcare professionals, researchers, and childcare professionals decided the questions included in this questionnaire as part of the core outcome set process. This means the questions have been decided by people, including parents like you, to help us best measure how people feed their babies.

### Outcome measurement

The primary outcome of interest is the proportion of questionnaire completion. Individual item response rates will also be assessed in terms of completion of questions on infant feeding outcomes, and other outcomes such as healthcare utilization and parent well-being. This is because the SWAT Intervention text specifically refers to infant feeding, and so this study will examine whether the intervention influenced completion of these outcomes specifically.

A secondary outcome of interest is participant attitudes about questionnaire completion. Data on participant attitudes will be collected via questions included at the end of the CHErIsH questionnaire; the CHErIsH questionnaire can be completed online, in-person, or by phone based on participant preference, and so the SWAT questions can be similarly completed. Quantitative data for participant attitudes will be collected for all participants using the following two questionnaire items, which are rated from on a 5-point Likert scale from ‘strongly disagree’ to ‘strongly agree’:

1. The infant-feeding related questions in the questionnaire were useful for gaining insight into how you feed your child.
2. The infant-feeding related questions in the questionnaire were appropriate for gaining insight into how you feed your child

Participants in the SWAT Intervention group will also be asked the following two questions, the first of which is closed-ended and rated from on a 5-point Likert scale from ‘strongly disagree’ to ‘strongly agree’. The second SWAT Intervention question is a single open-ended question that allows participants to describe in their own words how the information on COS influenced their completion of the questionnaire. Both questions are as follows:

3. The information provided about Core Outcome Sets (COSs) influenced my completion of the questionnaire.
4. How did the information about Core Outcome Sets (COSs) influence your completion of the questionnaire?

### Analysis

*Quantitative analysis*: All questionnaire data will be entered into SPSS Version 24 software. Questionnaire response rates will be calculated for each of the SWAT Intervention and SWAT Comparator including proportion of completion of the CHErIsH questionnaire and individual item response rates. Chi squared tests will compare the proportion of the questionnaire completed for the two conditions. Potential differences between participant baseline characteristics (age, sex, education) will also be examined and should differences be observed, these will be controlled for using logistic regression.

Data from the SWAT Intervention group in response to the question 3 will be descriptively summarised in terms of participants’ mean attitude rating, standard deviation and range of ratings. As this data is only collected from the SWAT Intervention, inferential statistics will not be conducted.

*Qualitative analysis*: Responses to the open-ended question will be entered into NVivo 12 for qualitative data management and will be analysed using thematic analysis following Braun and Clarke (2006) guidelines. This will involve an iterative process of reading and re-reading the data, developing initial line codes, followed by categorisation and development of themes. However, if there is insufficient detail in the open-ended responses then they will be examined narratively.

### Dissemination

Findings of this study will be disseminated via peer-reviewed publications and conference presentations. Anonymised data will be made available on an open access repository.

### Study status

This study within a trial will begin in January 2019, when the CHErIsH feasibility study begins.

## Discussion

The SWAT embedded within the CHErIsH feasibility trial is an important step for evaluating additional potential benefits of COSs in trial methodology beyond the benefits of COSs for evidence synthesis ( Williamson *et al*., 2012). Evidence suggests that well-informed participants are more willing to participate and engage in health research ( Ellis *et al*., 2002; Treweek *et al*., 2018b). Increasing participant knowledge of different aspects of trial processes therefore has the potential to increase response rates and minimise attrition in trials. Specifically, informing trial participants about the development and measurement of a COS has the potential to increase participant response rates in a number of ways. The first is through provision of information to increase participant knowledge. The second is through highlighting subjective norms in terms of outcomes of importance in trials where a COS has been developed by stakeholder groups representative of the participant group; subjective norms can influence behavioural intentions and subsequent behaviour. The third is via use of credible sources, for instance in the form of perceived expert stakeholders in COS development.

Some weaknesses of this SWAT need to be considered. For instance, recruitment of participants for the SWAT is dependent on numbers recruited to the larger CHErIsH trial. Precise and accurate conclusions can only be drawn from an appropriate sample size, therefore insufficient sample size will adversely impact statistical power to detect a difference between the two conditions ( Nayak, 2010). Inclusion of additional SWAT questions at the end of the CHErIsH trial questionnaire may also increase participant burden, which could impact on questionnaire completion rates. However, care was taken by the research team to develop questions that are as brief as possible to minimise this, and these questions are presented at end of CHErIsH questionnaire such that they their presence will potentially have minimal impact on completion. A strength of this SWAT is that it is embedded within a larger trial conducted within an engaged primary care practice. Recruitment will be conducted by post and in-person in the primary care practice, thus maximising and utilizing all avenues for participant recruitment and engagement. This SWAT uses a mixed-methods approach to data collection, with closed ended questions allowing for evaluation of participants’ attitudes towards questionnaire completion in both conditions. The open-ended question allows participants in the SWAT Intervention to describe in their own words how COS information influenced their completion of the questionnaire. This approach will facilitate an understanding of whether and how the SWAT intervention worked ( Farquhar *et al*., 2011). A further strength is that this SWAT was designed following best practice SWAT guidelines ( Treweek *et al*., 2018a), particularly in terms of appropriate use of randomisation and appropriate planning of analysis and implementation. Furthermore, this SWAT draws on mechanisms of behaviour including BCTs ( Michie *et al*., 2013) and the theory of planned behaviour ( Ajzen, 1991). These theoretical underpinnings ensure that the proposed rationale of this SWAT moves beyond simply thinking that the information alone will influence questionnaire completion rates ( Nyman *et al*., 2018). By examining whether informing participants of the use of an infant feeding COS influences questionnaire response rates and questionnaire completion, findings of the SWAT will significantly contribute to the literature on strategies for maximising participant response rates in trials.

## Ethics approval and consent to participate

The research was approved in Ireland by the Clinical Research Ethics Committee of the Cork Teaching Hospitals, UCC.

On commencement of the trial, all participants will provide signed consent for participation in the study and publication of results.

## Data availability

### Underlying data

Currently there are no available data associated with this article as the feasibility trial has yet to commence.

### Extended data

Open Science Framework: COS SWAT (The SWAT Questionnaire), https://doi.org/10.17605/OSF.IO/VHJS4 ( Matvienko-Sikar, 2018).

Data are available under the terms of the Creative Commons Attribution 4.0 International license (CC-BY 4.0).

## References

[ref-1] Ajzen I: The theory of planned behavior. *Organ Behav Hum Decis Process.* 1991;50(2):179–211. 10.1016/0749-5978(91)90020-T

[ref-2] Al-Shahi Salman R Beller E Kagan J: Increasing value and reducing waste in biomedical research regulation and management. *Lancet.* 2014;383(9912):176–85. 10.1016/S0140-6736(13)62297-7 24411646 PMC3952153

[ref-3] Brueton VC Tierney JF Stenning S: Strategies to improve retention in randomised trials: a Cochrane systematic review and meta-analysis. *BMJ Open.* 2014;4(2):e003821. 10.1136/bmjopen-2013-003821 24496696 PMC3918995

[ref-4] COMET: COMET plain language smmary.2018 Reference Source

[ref-5] Edwards PJ Roberts I Clarke MJ: Methods to increase response to postal and electronic questionnaires. *Cochrane Database Syst Rev.* 2009; (3):MR000008. 10.1002/14651858.MR000008.pub4 19588449 PMC8941848

[ref-6] Ellis PM Butow PN Tattersall MH: Informing breast cancer patients about clinical trials: a randomized clinical trial of an educational booklet. *Ann Oncol.* 2002;13(9):1414–1423. 10.1093/annonc/mdf255 12196367

[ref-7] Ellis PM Butow PN Tattersall MH: Randomized clinical trials in oncology: understanding and attitudes predict willingness to participate. *J Clin Oncol.* 2001;19(15):3554–3561. 10.1200/JCO.2001.19.15.3554 11481363

[ref-8] Ellis PM Dowsett SM Butow PN: Attitudes to randomized clinical trials amongst out-patients attending a medical oncology clinic. *Health Expect.* 1999;2(1):33–43. 10.1046/j.1369-6513.1999.00028.x 11281873 PMC5061398

[ref-9] Farquhar MC Ewing G Booth S: Using mixed methods to develop and evaluate complex interventions in palliative care research. *Palliat Med.* 2011;25(8):748–757. 10.1177/0269216311417919 21807749

[ref-10] Fewtrell MS Kennedy K Singhal A: How much loss to follow-up is acceptable in long-term randomised trials and prospective studies? *Arch Dis Child.* 2008;93(6):458–461. 10.1136/adc.2007.127316 18495909

[ref-11] Gardner W Kelleher KJ: Core Quality and Outcome Measures for Pediatric Health. *JAMA Pediatr.* 2017;171(9):827–828. 10.1001/jamapediatrics.2017.1685 28692714

[ref-12] Gillies K Entwistle VA: Supporting positive experiences and sustained participation in clinical trials: looking beyond information provision. *J Med Ethics.* 2012;38(12):751–6. 10.1136/medethics-2011-100059 22875981

[ref-32] Matvienko-Sikar K: COS SWAT.2018 10.17605/OSF.IO/VHJS4

[ref-13] Matvienko-Sikar K Byrne M Kelly C: Developing an infant feeding core outcome set for childhood obesity prevention. *Lancet.* 2018a;392:S59 10.1016/S0140-6736(18)32887-3

[ref-14] Matvienko-Sikar K Byrne M Kelly C: Development of an infant feeding core outcome set for childhood obesity interventions: study protocol. *Trials.* 2017a;18(1):463. 10.1186/s13063-017-2180-4 29017519 PMC5634841

[ref-15] Matvienko-Sikar K Griffin C McGrath N: Developing a core outcome set for childhood obesity prevention: A systematic review. *Matern Child Nutr.* 2018b;e12680. 10.1111/mcn.12680 30136417 PMC7199036

[ref-16] Matvienko-Sikar K Toomey E Delaney L: Effects of healthcare professional delivered early feeding interventions on feeding practices and dietary intake: A systematic review. *Appetite.* 2018;123:56–71. 10.1016/j.appet.2017.12.001 29225141

[ref-17] Michie S Richardson M Johnston M: The behavior change technique taxonomy (v1) of 93 hierarchically clustered techniques: building an international consensus for the reporting of behavior change interventions. *Ann Behav Med.* 2013;46(1):81–95. 10.1007/s12160-013-9486-6 23512568

[ref-18] Nayak BK: Understanding the relevance of sample size calculation. *Indian J Ophthalmol.* 2010;58(6):469–70. 10.4103/0301-4738.71673 20952828 PMC2993974

[ref-19] Nyman SR Adamczewska N Howlett N: Systematic review of behaviour change techniques to promote participation in physical activity among people with dementia. *Br J Health Psychol.* 2018;23(1):148–170. 10.1111/bjhp.12279 28980370

[ref-20] Parveen S Islam MS Begum M: It's not only what you say, it's also how you say it: communicating nipah virus prevention messages during an outbreak in Bangladesh. *BMC Public Health.* 2016;16:726. 10.1186/s12889-016-3416-z 27495927 PMC4974711

[ref-21] Redfern J Santo K Coorey G: Factors Influencing Engagement, Perceived Usefulness and Behavioral Mechanisms Associated with a Text Message Support Program. *PLoS One.* 2016;11(10):e0163929. 10.1371/journal.pone.0163929 27741244 PMC5065147

[ref-22] Redsell SA Edmonds B Swift JA: Systematic review of randomised controlled trials of interventions that aim to reduce the risk, either directly or indirectly, of overweight and obesity in infancy and early childhood. *Matern Child Nutr.* 2016;12(1):24–38. 10.1111/mcn.12184 25894857 PMC5029770

[ref-23] Sahlqvist S Song Y Bull F: Effect of questionnaire length, personalisation and reminder type on response rate to a complex postal survey: randomised controlled trial. *BMC Med Res Methodol.* 2011;11:62. 10.1186/1471-2288-11-62 21548947 PMC3110121

[ref-24] Schulz KF Grimes DA: Sample size slippages in randomised trials: exclusions and the lost and wayward. *Lancet.* 2002;359(9308):781–785. 10.1016/S0140-6736(02)07882-0 11888606

[ref-25] Scott P Edwards P: Personally addressed hand-signed letters increase questionnaire response: a meta-analysis of randomised controlled trials. *BMC Health Serv Res.* 2006;6:111. 10.1186/1472-6963-6-111 16953871 PMC1574304

[ref-26] Treweek S Bevan S Bower P: Trial Forge Guidance 1: what is a Study Within A Trial (SWAT)? *Trials.* 2018a;19(1):139. 10.1186/s13063-018-2535-5 29475444 PMC5824570

[ref-27] Treweek S Pitkethly M Cook J: Strategies to improve recruitment to randomised trials. *Cochrane Database Syst Rev.* 2018b;2: MR000013. 10.1002/14651858.MR000013.pub6 29468635 PMC7078793

[ref-28] Webbe J Modi N Gale C: Core Quality and Outcome Measures for Pediatric Health. *JAMA Pediatr.* 2018;172(3):299–300. 10.1001/jamapediatrics.2017.5050 29297042

[ref-29] Whitford H Hoddinott P Amir LH: Routinely collected infant feeding data: Time for global action. *Matern Child Nutr.* 2018;14(4):e12616. 10.1111/mcn.12616 29781212 PMC6866076

[ref-30] Williamson PR Altman DG Bagley H: The COMET Handbook: version 1.0. *Trials.* 2017;18(Suppl 3):280. 10.1186/s13063-017-1978-4 28681707 PMC5499094

[ref-31] Williamson PR Altman DG Blazeby JM: Developing core outcome sets for clinical trials: issues to consider. *Trials.* 2012;13:132. 10.1186/1745-6215-13-132 22867278 PMC3472231

