# Supplementary material for: Influence of providing information to participants about development of trial outcomes on response rates and attitudes to questionnaire completion: Protocol for a study within a trial
Source: HRB Open Res. 2019 May 20;2:2. Originally published 2019 Jan 9. [Version 2] doi: 10.12688/hrbopenres.12895.2 (PMC6973521; doi:10.12688/hrbopenres.12895.2)
Supplement: Peer review history

# Peer review: Reviewer response for version 2

**DOI:** 10.21956/hrbopenres.13989.r26602

**Published:** 2019-06-03

**Authors:** Shaun P. Treweek

**Affiliations:** 1 Health Services Research Unit (HSRU), Institute of Applied Health Sciences, School of Medicine, Medical Sciences and Nutrition, University of Aberdeen, Aberdeen, UK

I confirm that I have read this submission and believe that I have an appropriate level of expertise to confirm that it is of an acceptable scientific standard.

# Peer review: Reviewer response for version 1

**DOI:** 10.21956/hrbopenres.13964.r26467

**Published:** 2019-02-06

**Authors:** Shaun P. Treweek, Beatriz Goulao, Karen Innes

**Affiliations:** 1 Health Services Research Unit (HSRU), Institute of Applied Health Sciences, School of Medicine, Medical Sciences and Nutrition, University of Aberdeen, Aberdeen, UK; 2 Health Services Research Unit (HSRU), Institute of Applied Health Sciences, School of Medicine, Medical Sciences and Nutrition, University of Aberdeen, Aberdeen, UK

SWATs such as this are a good way to improve the evidence base for trial process decision-making, so we welcome this protocol. We do have a few comments, all of which are about reporting clarity.

**General**

 1.  We found the ‘I1 condition’ and ‘I2 condition’ terminology confusing. ‘Condition’ in particular made us think about an illness or disease. We would suggest ‘SWAT intervention’ and ‘SWAT comparator’ but the authors might think of something better. Either way, we’d like to see a change from ‘I1 condition’ and ‘I2 condition’ to something else.

 2.  We would have liked to have seen the information about COS that is presented to participants as part of the SWAT, together with the comparator text. Knowing this will make it easier for others to replicate the evaluation of the SWAT intervention. There was a statement suggesting that this was in the extended data but we didn’t find it. Sorry if there was a problem with the information we received rather than what you submitted.  

**Abstract**

 1.  The abstract says that response rates will be measured as proportion of full questionnaire completion – what happens to questionnaire that are partially completed? Are they counted as non-responses? 

**Methods**

 1.  It would be good to know a few things about the host trial: 1) how many are involved in the feasibility trial?  2) How many items are collected on the host trial questionnaire?

 2.  The paper is missing a clear definition of the outcomes it will measure to assess the effect of the SWAT intervention. In particular what is the primary outcome and when will it be measured? We began to speculate as to whether the measurement was only at baseline, which didn’t seem to make sense. What we’d like to see is a clear indication of when the outcome assessment for the SWAT will be done. 

 3.  We didn’t find Figure 1 helpful. It suggests that both groups are receiving the SWAT intervention but the text in the manuscript says ‘Information about the COS will be provided to a random sample of half of all participants in a brief paragraph..’, which suggests that not everyone gets it. The diagram would be better as a more standard CONSORT-esque figure with the timings as you work you way down the figure linked to the timing of outcome assessment.

 4.  The 4-item ‘SWAT questionnaire’ confused us a bit because not all questions are asked of all participants. Only questions 3 and 4 are unique to the SWAT intervention, we’re guessing that questions 1 and 2 are not specifically linked to the COS text that forms the SWAT intervention. Why not have questions 1 and 2 (common to both arms) as part of the main questionnaire and the SWAT questionnaire is then just questions 3 and 4? We also weren’t sure how the items on the questionnaire related to the outcomes of the SWAT. They are not response rates so we guess that they will be linked to attitudes. It would be good to know how this work will be done with the 5-point Likert scales and how the free-text in question 4 will be handled.  

 5.  Where is the SWAT outcome assessment done? Is it a postal questionnaire, linked to a visit for the host trial, or something else?

 6.  We weren’t clear why the researchers were measuring response rates for full questionnaire and individual items and which of these is considered the most important?  And we assumed (but weren’t sure) that the completion of the host trial’s primary outcome was the trial outcome that you were concentrating on with regard to  increasing response rates. Or is it all of the host trial outcomes? 

 7.  It would be useful to see a statement about how many people you think will be involved in the SWAT.  This will be limited by the size of the Cherish trial so we’re not asking for a sample size calculation, just an indication of how many people are likely to be involved.

 8.  Can you say a bit more about why (and what) you will adjust for when looking at baseline imbalance. Any differences will be due to chance if the randomisation works though if the sample is small, it is true that these may lead to under or over-estimates of effect if the differences are things that affect your outcomes.  

**Discussion**

 1.  The SWAT does involve extra data collection (not all SWATs do) and we could speculate that this could reduce response rates to the host trial questionnaire. Could the authors comment on this?

We confirm that we have read this submission and believe that we have an appropriate level of expertise to confirm that it is of an acceptable scientific standard, however we have significant reservations, as outlined above.

# Peer review: Reviewer response for version 1

**DOI:** 10.21956/hrbopenres.13964.r26468

**Published:** 2019-01-18

**Authors:** Susanna Dodd

**Affiliations:** 1 Department of Biostatistics, Institute of Translational Medicine, University of Liverpool, Liverpool, UK

This is a well-written protocol describing a SWAT which will provide useful information regarding the impact of COS knowledge on participant engagement in questionnaire completion. I am happy to recommend that this protocol should be indexed, providing the following issues are addressed:

1. I have only one major comment relating to the apparent omission of details relating to the sample size target. Please could the authors provide details of the recruitment target, along with justification for the required sample size.
2. I have a few minor suggestions for improving the grammar and general flow of the article:
  Abstract Methods: 2nd sentence: "consisting" should be replaced with "consists"
  Introduction: 1st paragraph: 2nd line:
  "increases" should be replaced with "increase"
  "reduces" should be replaced with "reduce"
  Introduction: 4th paragraph:
  2nd line: Add "how" before "questionnaires"
  11th line: Move "however" to after "has not"
  Introduction: last paragraph: 7th line:
  Add apostrophe after "participants"
  Methods:
  SWAT section:
  1st paragraph: 11th sentence: Change "dependent" to "depending"
  2nd paragraph: 1st sentence: Change this sentence to
  "The SWAT informational intervention will be provided to those randomised to the l1 condition and will consist of a brief paragraph..."
  Analysis: Quantitative analysis:
  Clarify the final sentence by adding "using logistic regression" after "controlled for".
  Discussion: 1st paragraph:
  2nd line: Remove "more" before "well-informed"
  7th line: Add "has been" after "where a COS"
  Discussion: 2nd paragraph:
  7th line: Add apostrophe at the end of "participants"
  11th line: Remove comma after "including"
  14th line: Replace "Through" with "By"; replace "if" with "whether"; repace "effects" with "affects"
  Data availability: change "is" to "are"

I confirm that I have read this submission and believe that I have an appropriate level of expertise to confirm that it is of an acceptable scientific standard.
